# Therapeutic effect of ginseng: a non-antibiotic approach to preventing pathogenic bacterial infections and restoring gut microbiota composition

**DOI:** 10.3389/fphar.2026.1866260

**Published:** 2026-09-08

**Authors:** Seong-Hyeon Kim, Changhan Lee

**Affiliations:** Department of Biological Sciences, Ajou University, Suwon, Republic of Korea

**Keywords:** acidic polysaccharides, gingseng, ginsenoside, gut microbiota, pathogenic bacterial infection

## Abstract

Human health is influenced by various factors, including bacteria that surround us ubiquitously. Bacteria constituting normal microbiota of various human body niches, primarily the gut, can confer beneficial effects by aiding food digestion, improving nutrient production, and preventing pathogen colonization. However, bacteria can also have harmful effects as pathogens, especially when the immune system is compromised due to various diseases. In particular, secondary or co-infections caused by pathogenic bacteria have been shown to significantly contribute to illness severity and increased mortality rates. Ginseng, an herb widely used for various medicinal purposes in Asia, including the treatment of cancer and immune-related diseases, has shown substantial effects in modulating the bacterial composition of the human body, including protecting human health against various pathogenic bacterial infections. Furthermore, recent studies have also demonstrated that ginseng can promote the restoration of disrupted gut microbiota composition, caused by various reasons such as intestinal inflammation, obesity, antibiotics-associated diarrhea, colitis and so on. Therefore, this review comprehensively discusses the effects of ginseng on pathogenic bacteria, as well as the recent findings on the impact of ginseng on gut microbiota imbalances caused by various factors.

## Graphical Abstract

**Figure:**
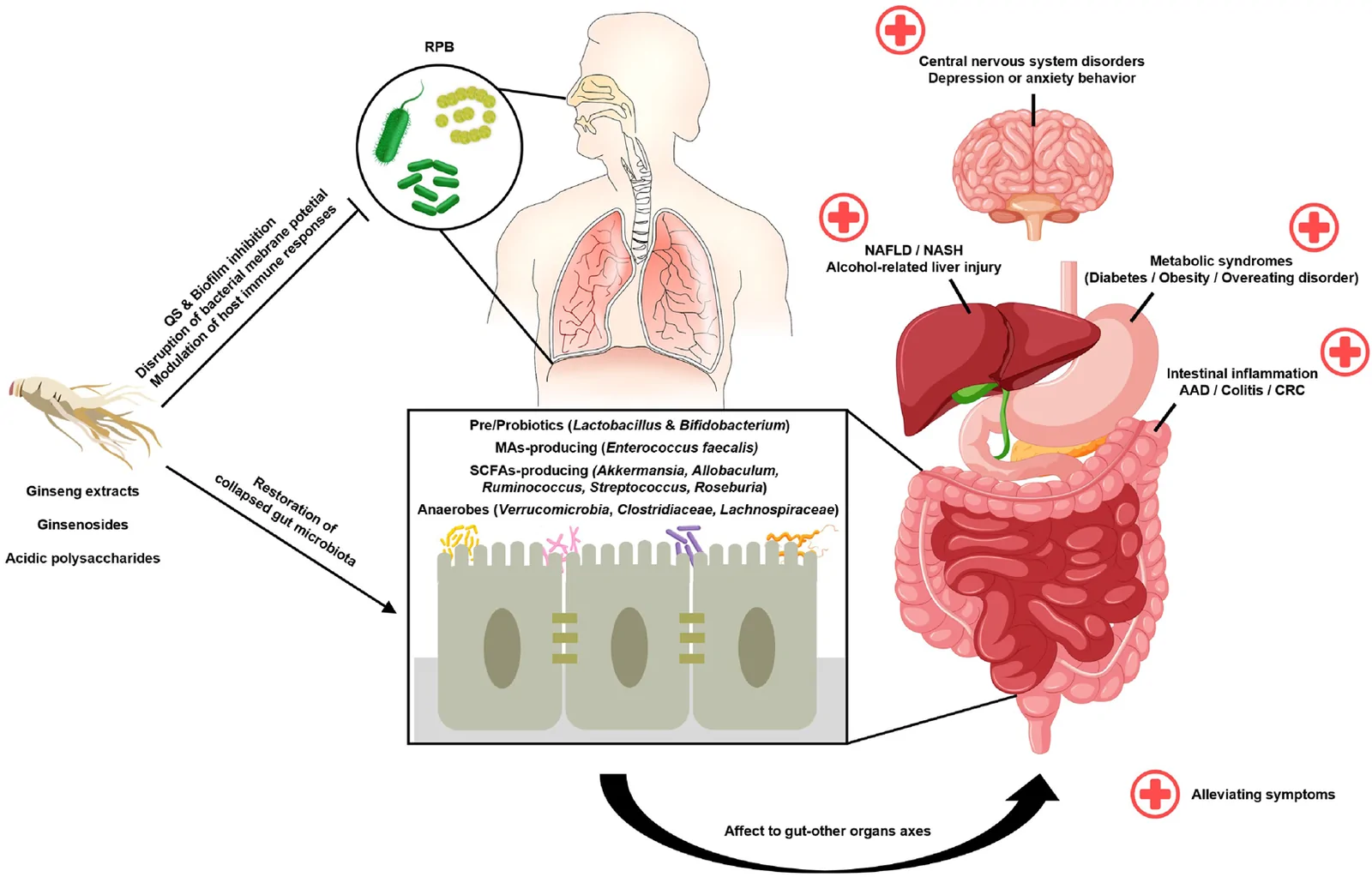

## 1 Introduction

Bacteria are single-celled organisms that are ubiquitously found in every niche on Earth, including within all living organisms. Bacteria demonstrate remarkable adaptability to thrive across varied environments and host conditions. Their ability to respond to environmental changes, often mediated through gene expression, allows them to fine-tune their adaptations. This diversity extends across different genera, species, and strains, each equipped with distinctive features tailored for survival and, in some cases, virulence.

Humans have a symbiotic relationship with bacteria. For instance, commensal gut microbiota is involved in the biotransformation of nutrients and can prevent colonization of pathogenic bacteria. They can also produce essential metabolites such as vitamins and short-chain fatty acids (SCFAs) (Lange et al., 2016). However, bacteria can also severely damage human health through chronic or acute infections. Secondary infections or co-infections involving respiratory pathogenic bacteria have been shown to significantly exacerbate disease severity and increased mortality, particularly in the context of viral respiratory illnesses such as influenza and COVID-19 (Mitsi et al., 2022; Rhoades et al., 2021; Lee et al., 2022; Liu et al., 2021). Moreover, the emergence of antibiotic resistance in bacteria poses a critical threat to human health (Lange et al., 2016; Kim and Yang, 2018).

Ginseng, a medicinal herb traditionally used in East Asian countries such as China, Japan, and Korea, is renowned for its diverse effects, including antimicrobial, anticancer, and immunomodulatory properties (Ratan et al., 2021). The principal component responsible for these effects is ginsenoside, which varies among different ginseng species and preparations (Kim et al., 2017). Given the current focus on promoting or controlling the growth of specific bacteria, extensive research has been conducted on the development of antibiotics, probiotics, and other interventions. Notably, attention has been paid to effects of ginseng on bacteria. This review summarizes effects and mechanisms of ginseng as a promising non-antibiotic therapeutic adjuvant for preventing respiratory pathogenic bacterial infections and restoring disrupted microbiota composition. How its complex effects, stemming from various compounds, contribute to these outcomes is discussed.

## 2 Ginseng

**FIGURE 1:**
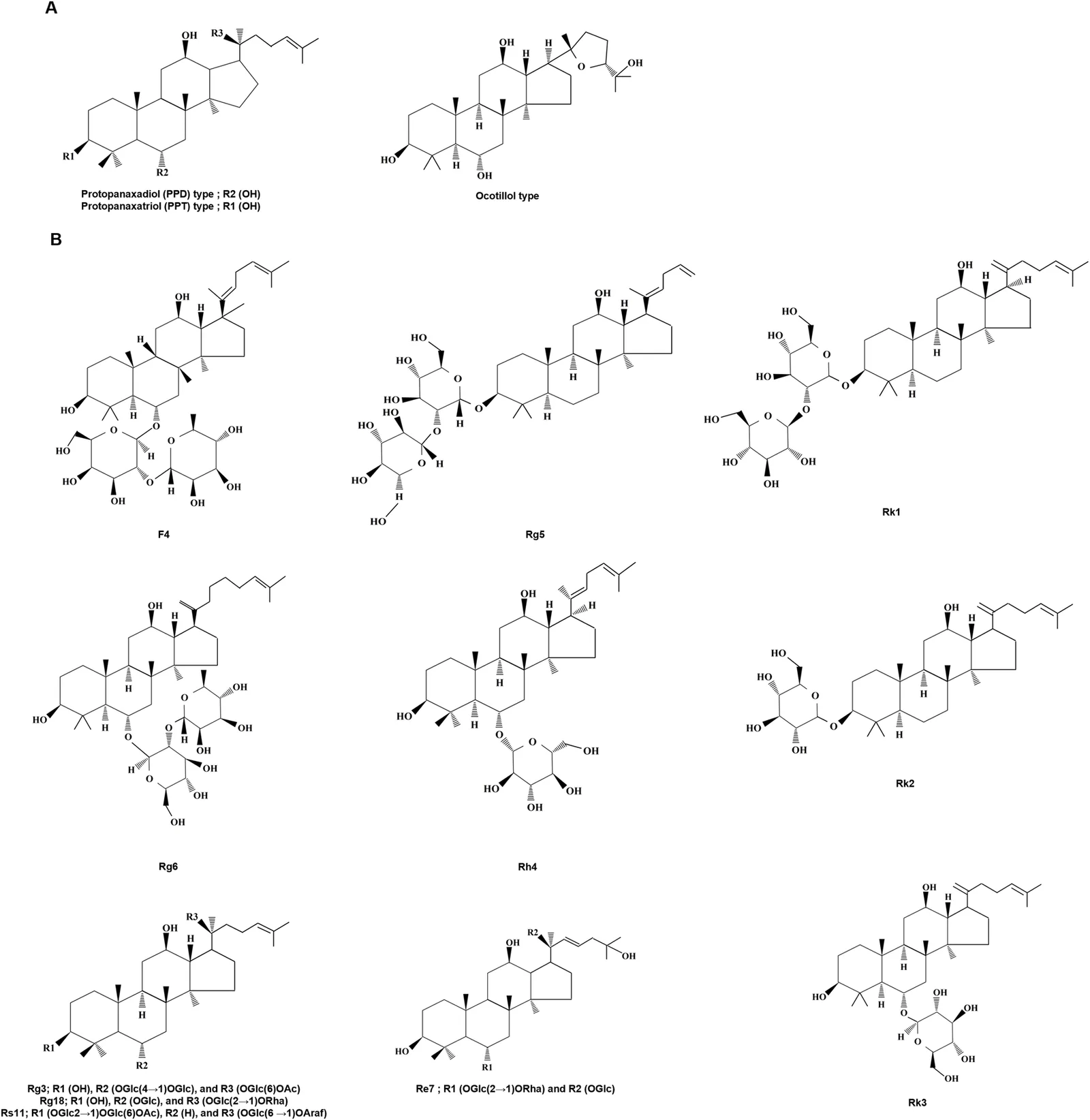
Structures and classification of ginsenosides known to possess antibacterial effects. **(A)** Structures of PPD and PPT type ginsenosides. Main types of antibacterial ginsenosides are PPD, PPT, and ocotillol type ginsenosides. PPD and PPT type ginsenosides are based on a dammarane backbone. Ocotillol type ginsenosides have tetracyclic triterpenoid and tetrahydrofuran ring at C20 position. **(B)** Structures of other types of ginsenosides. PPD: Protopanaxadiol; PPT: Protopanaxatriol.

Ginseng is a naturally growing herb in Northeast Asia (China, Japan, Korea, and Russia) and North America. To date, three major types of ginsengs, including *Panax ginseng* Meyer (Korean ginseng), *Panax notoginseng* (Chinese ginseng), and *Panax quinquefolius* (American ginseng), are cultivated commercially. Ginseng is known to have potential therapeutic effects against various diseases, such as antimicrobial, anti-oxidative, anti-inflammatory, anti-tumor, anti-obesity, anti-diabetes, and anti-aging activities (Ratan et al., 2021). The core pharmacological compound is ginsenoside (dammarane backbone: protopanaxadiol (PPD); additional -OH group at C6 of dammarane backbone: protopanaxatriol (PPT) type; tetracyclic terpenoid with tetrahydrofuran (5-membered epoxy) ring at C20: ocotillol type; and others), which is produced through isoprenoid pathways (Figures 1A,B) (Kim et al., 2017). It also contains adjuvant compounds, such as acidic polysaccharides (Lü et al., 2009). Therefore, controlling techniques to preserve ginsenosides are crucial for maintaining a consistent efficacy of ginseng.

To understand pharmacological functions of ginseng and to control the quality of ginsenosides during the production or processing of ginseng, various studies, including genomics, transcriptomics, and metabolomics, have been recently conducted. The genome of *P. ginseng* Meyer was analyzed and constructed through *de novo* genome assembly and the Ginseng Genome Database was opened to provide access to ginseng genomes, transcriptomes, and annotated functional genes (Jayakodi et al., 2018; Kim et al., 2018a). Several studies have elucidated that the patterns of ginsenosides, polysaccharides, and amino acids abundance in ginseng vary widely in accordance with its agricultural season (Lee et al., 2020; Song et al., 2019).

The secondary process of administered ginsenosides in the gut is also important. Administered ginsenosides are metabolized by gut microbiota to subordinate ginsenosides such as compound K (CK) for absorption by host cells through deglycosylation, oxygenation, and hydration processes (Mancuso and Santangelo, 2017). Although accurate metabolic pathways of ginsenosides by gut microbiota are currently unknown, numerous studies have demonstrated that *Bifidobacterium, Lactobacillus, Fusobacterium, Eubacterium,* and *Bacteroides* are major ginsenosides-metabolizing gut microbiota (Kim et al., 2017; Bae et al., 2000; Bae et al., 2002a; Bae et al., 2002b; Bae et al., 2005; Quan et al., 2013; Zhang et al., 2021a).

Because co- and secondary infections are often driven by opportunistic bacterial pathogens, such as *Pseudomonas aeruginosa* and *Staphylococcus aureus*, it is important to evaluate whether ginseng-derived compounds—either in their native forms or after microbiota-mediated transformation—can directly inhibit these bacteria or attenuate their virulence. In the following section, we summarize current evidence for the activity of ginseng against clinically relevant pathogens, focusing on growth inhibition, regulation of biofilm formation and quorum sensing, interference with adhesion, and modulation of host immune responses.

## 3 Effects of ginseng on pathogenic bacterial infection

### 3.1 Ginseng extracts

Aqueous extracts of ginseng are able to regulate motility mode, biofilm formation, and membrane permeability. Twitching and swimming motility of *P*. *aeruginosa* PAO1 were increased while the swarming motility was decreased after treatment with *P. ginseng* aqueous extracts (Wu et al., 2011). Notably, *P. ginseng* aqueous extracts disrupted 7-day-old mature *P. aeruginosa* PAO1 biofilms within 24 h exposure at a concentration of 0.5% (Wu et al., 2011). Brazilian ginseng extracts decreased 18.86% of *Klebsiella pneumoniae* biomass and 72.38% of *K. pneumoniae* biofilm formation (Paula-Ramos et al., 2016). Flower extracts (FE) of *P. ginseng* and root extracts (RE) of *P. notoginseng* inhibited QS signals in *P. aeruginosa* PAO1 (FE: 50%; RE: 32%) (Koh and Tham, 2011). Furthermore, *P. ginseng* whole extracts decreased LasAB protease system production and N-acyl homoserine lactone (AHL) biosynthesis in *P. aeruginosa* PAO1, although it cannot inhibit the growth of *P. aeruginosa* PAO1 (Song et al., 2010). Heated Korean ginseng extracts inhibited the growth of *S. aureus* by collapsing bacterial membrane potential (Ahn et al., 2006).

In terms of host immune response, the activity of polymorphonuclear leukocytes (PMNs) was increased while lung pathology was decreased in rats treated with ginseng extracts (Wu et al., 2011). IgA level was increased in the lung and IgG subclasses (IgG1 to IgG2; Th-2-like to Th-1-like response) were changed in rat and mouse (Wu et al., 2011; Song et al., 2018). In addition, lung immune responses were enhanced by upregulated IFN-γ or TNFα and downregulated IL-4 in ginseng treated mouse (Song et al., 2010). Pre-treatment with Korean red ginseng (KRG) extracts reduced levels of acute inflammation-related cytokines and the production of reactive oxygen species (ROS) in mice by inhibiting nitric oxide generation in host cells. In addition, the PI3K/AKT signaling pathway and phagocytosis are upregulated upon pre-treatment with KRG extracts (Nguyen et al., 2015; Lee et al., 2019). Ginseng extracts and purified ginsenoside Rb1 modulated immune responses in cattle and mice *via* enhanced phagocytosis activity, lymphocyte or monocyte proliferation, and regulation of cytokines against *S*. *aureus* infection (Hu et al., 2001; Silvestrini et al., 2017; Beccaria et al., 2018).

### 3.2 Ginsenosides

Ginsenosides have specific ring-structured backbone structures. They are classified based on their ring structures or presence of active groups. PPD, PPT, and ocotillo types of ginsenosides are representative antibacterial compounds of ginseng (Tables 1–3) (Luong et al., 2021). Antibacterial effects of these ginsenosides are determined based on hydrophilicity of specific active groups such as glycosyl group, aglycone, or sugar bonds, and its configuration (Xue et al., 2020). When hydrophile-lipophile balance (HLB) value rises, the antibacterial effect of ginsenosides generally increases (Xue et al., 2020). In addition, less polar ginsenosides (PPT) generally exhibit higher pharmacological activity than polar ginsenosides (Xue et al., 2020). The correlation (R^2^) between the HLB value of ginsenoside and its antibacterial effect (MIC value) has been calculated as 0.747 for *Fusobacterium nucleatum* and 0.544 for *Bacillus cereus* (Na et al., 2018). Ginsenoside 20(S)-Rh2 enhanced the accumulation of ciprofloxacin and levofloxacin in *S. aureus* cells by inhibiting efflux transporters, which can lead to elimination of antibiotic-resistant *S. aureus* strains (Zhang et al., 2014; Chen et al., 2021). Several pre-heat-treated ginsenoside-enriched fractions, especially ginsenoside Rg5, inhibited the growth of *P. gingivalis* by breaking down bacterial membrane integrity (Xue et al., 2017). Ginsenosides, as amphipathic molecules, form micelle structures and bind with sterols of bacterial cell membrane and promote derangement of biofilm construction (Xue et al., 2020; Böttger and Melzig, 2013).

**TABLE 1:** Reported antibacterial effects of PPD and PPT type ginsenosides.

Type	Ginsenoside	R1 at C3	R2 at C6	R3 at C20	Target bacterial species	Ref.
PPD	Protopanaxadiol	OH	H	OH	*H. pyroli*	(Jee et al., 2008)
	Rb_1_	OGlc (1→2)Glc	H	OGlc (1→6)Glc	*S. aureus* ^*^	(Sung and lee, 2008)
	Rc	OGlc (1→2)Glc	H	OAraf (1→6)Glc	*S. aureus* ^*^	
	Rd	OGlc (1→2)Glc	H	OGlc	*S. aureus* ^*^	
	Rb_2_	OGlc (1→2)Glc	H	OArap (1→6)Glc	*S. aureus* ^*^ *, S. captis* *Salmonella epidermidis, K. pneumoniae*	(Sung and lee, 2008; Battinelli et al., 1998)
	Rh_2_	OGlc	H	OH	*S. aureus, S. captis, K. pneumoniae, F. nucleatum, C. perfringens, P. gingivalis, L. ivanovii, P. aeruginosa, S. epidermidis, B. cereus, Streptococcus mutans, Streptococcus sobrinus, Streptococcus sanguinis*	(Xue et al., 2020; Zhang et al., 2014; Chen et al., 2021; Xue et al., 2017; Sung and lee, 2008; Cao et al., 2019)
	Rg_3_	OGlc (1→2)Glc	H	OH	*E. coli, S. aureus, F. nucelatum, C. perfringens, P. gingivalis, P. aeruginosa, S. epidermidis, B. cereus*	(Xue et al., 2020; Xue et al., 2017; Lee et al., 2015)
PPT	Protopanaxatriol	OH	OH	OH	*H. pyroli*	(Bae et al., 2001)
	Re	OH	ORha (1→2)Glc	OGlc	*S. aureus* ^*^ *, S. captis, S. epidermidis, K. pneumoniae*	(Sung and lee, 2008; Battinelli et al., 1998)
	Rf	OH	OGlc (1→2)Glc	OH	*S. aureus* ^#^	(Sung and lee, 2008)
	Rg_1_	OH	OGlc	OGlc	*S. aureus, S. epidermidis, B. cereus, K. pneumoniae*	(Xue et al., 2020; Sung and lee, 2008)
	Rg_2_	OH	ORha (1→2)Glc	OH	*S. aureus, C. perfringens, F. nucleatum, P. gingivalis, P. aeruginosa, S. epidermidis, B. cereus, P. acnes* ^*^	(Xue et al., 2020; Sung and lee, 2008; Battinelli et al., 1998)

(*: the antibacterial effect is attributed to the ginsenoside mixture) PPD: protopanaxadiol; PPT: protopanaxatriol.

**TABLE 2:** Reported antibacterial effects of ocotillol type ginsenosides.

Derivatives	Molecular formula	Target bacteria	Ref.
16a	*(20S, 24S)-Epoxy-3β-O-(2-nitrooxy acetyl)-dammarane-12β, 25-diol*	*S, aureus, B. subtilis*	(Bi et al., 2015)
16b	*(20S, 24S)-Epoxy-3β-O-(5-nitrooxy pentanoyl)-dammarane-12β, 25-diol*	*E. coli*	
16c	*(20S, 24S)-Epoxy-3β-O-(6-nitrooxy hexanoyl)-dammarane-12β, 25-diol*	*E. coli*	
17a	*(20S, 24R)-Epoxy-3β-O-(2-nitrooxy acetyl)-dammarane-12β, 25-diol*	*B. subtilis, E. coli*	
17c	*(20S, 24R)-Epoxy-3β-O-(6-nitrooxy hexanoyl)-dammarane-12β, 25-diol*	*S. aureus, B. subtilis, E. coli, P. aeruginosa, Acinetobacter baumanii*	
4	*(20S, 24)-Epoxy-24-carbonyl dammarane-3β, 12β-diol*	*S. aureus, B. subtilis, P. aeruginosa*	(Zhang et al., 2020)
4b	*(20S, 24)-Epoxy-24-carbonyl dammarane-3-one*	*B. subtilis, P. aeruginosa*	
4e	*(20S, 24)-Epoxy-3β-O-(2E, 4E-diene adipoyl)-24-carbonyl-dammarane-12β-ol*	*B. subtilis*	
4g	*(20S, 24)-Epoxy-3β-O-(3-carboxy-propionyl)-24-carbonyl-dammarane-12β-ol*	*S. aureus, B. subtilis*	
4h	*(20S, 24)-Epoxy-3β-O-(4-carboxy-butyryl)-24-carbonyl-dammarane-12β-ol*	*S. aureus, B. subtilis*	
4i	*(20S, 24)-Epoxy-3β-O-(2E-ene butyryl)-24-carbonyl-dammarane-12β-ol*	*B. subtilis*	
4j	*(20S, 24)-Epoxy-3β-O-(2-amino acetyl)-24-carbonyl-dammarane-12β-ol*	*S. aureus, B. subtilis, E. coli, P. aeruginosa*	
4k	*(20S, 24)-Epoxy-3β-O-(4-amino butyryl)-24-carbonyl-dammarane-12β-ol*	*S. aureus, B. subtilis, E. coli, P. aeruginosa*	
4L	*(20S, 24)-Epoxy-3β-O-(6-amino hexanoyl)-24-carbonyl-dammarane-12β-ol*	*S. aureus, B. subtilis, E. coli, P. aeruginosa*	
4m	*(20S, 24)-Epoxy-3β-O-(2-piperidine formyl)-24-carbonyl-dammarane-12β-ol*	*S. aureus, B. subtilis, E. coli, P. aeruginosa*	
4n	*(4S)-N-ethyl-4-((3S,8R,10R,12R,14R,17S)-3,12-dihydroxy-4,4,8,10,14-pentamethylhexadecahydro-1H-cyclopenta phenanthren-17-yl)-4-hydroxypentanamide*	*S. aureus*	
4p	*(4S)-N-butyl-4-((3S,8R,10R,12R,14R,17S)-3,12-dihydroxy-4,4,8,10,14-pentamethylhexadecahydro-1H-cyclppenta phenanthrene-17-yl)-4-hydroxypentanamide*	*S. aureus, E. coli, B. subtilis*	
3	*(20S, 24S)-Epoxy-dammarane-3β,12β,25-triol*	MRSA	(Zhou et al., 2013)
5	*(20S, 24R)-Epoxy-25-hydroxy-dammarane-3,12-dione*	MRSA	
16	*(20S, 24S)-Epoxy-12β-O-(2-carboxy benzoyl)-dammarane-3β,25-diol*	MRSA	
24	*(20S, 24R)-Epoxy-12β-O-(2-carboxy benzoyl)-dammarane-3β,25-diol*	MRSA	

MRSA: Meticillin-resistant *Staphylococcus aureus*.

**TABLE 3:** Reported antibacterial effects of other type ginsenosides.

Ginsenoside	Target bacterial species	Ref.
F4	*S. aureus, F. nucleatum, C. perfrigens, P. gingivalis, P. aeruginosa, S. epidermidis, B. cereus, P. acnes* ^*^	(Xue et al., 2020; Xue et al., 2017; Wang et al., 2013)
Rg5	*S. aureus, F. nucleatum, C. perfrigens, P. gingivalis, Listeria ivanovii, S. enteritidis, P. aeruginosa, S. epidermidis, B. cereus, S. captis, S. epidermidis, K. pneumoniae, P. acnes* ^*^	
Rg6	*S. aureus, F. nucleatum, C. perfrigens, P. gingivalis, P. aeruginosa, S. epidermidis, B. cereus, P. acnes* ^*^	
Rh4	*S. aureus, F. nucleatum, C. perfrigens, P. gingivalis, P. aeruginosa, S. epidermidis, B. cereus*	(Xue et al., 2020; Xue et al., 2017)
Rk1	*C. perfrigens, F. nucleatum, P. gingivalis, S. captis, S. epidermidis, K. pneumoniae, P. acnes* ^*^	(Xue et al., 2017; Wang et al., 2013)
Rk2	*S. aureus, F. nucleatum, C. perfrigens, P. gingivalis, L. ivanovii, S. enteritidis, P. aeruginosa, S. epidermidis, B. cereus*	(Xue et al., 2020)
Rk3	*S. aureus, F. nucleatum, C. perfrigens, P. gingivalis, L. ivanovii, S. enteritidis, P. aeruginosa, S. epidermidis, B. cereus*	
Rs3	*P. acnes* ^*^	(Wang et al., 2013)
Re7	*E. coli, S. aureus*	(Lee et al., 2015)
Rs11	*E. coli, S. aureus*	
Rg18	*E. coli, S. aureus*	

(*: the antibacterial effect is generated by ginsenoside mixture).

There are a significant number of reports that ginsenosides also affect to immune system of host cells by attenuation of cell signaling pathways, post-translational modification in host cells, and programmed cell death (PCD). Pre-treatment compound K rich fraction of KRG inhibited abnormal Toll-like receptor (TLR) signaling pathway and its downstream NF-κB and MAPK pathways (Yang et al., 2008; Yang et al., 2007). Ginsenoside Rb1/Rg3 treatment induced the production of neurotrophic factors and anti-apoptotic factors in brains of mice (Lee et al., 2018). Increased neurotrophic factors can trigger microglial activation and upregulate anti-apoptotic factors, leading to inhibition of microglial apoptosis by affecting the JAK2/STAT5 phosphorylation signaling pathway (Lee et al., 2018). Ginsenosides Rb1, Rg1, and Rg3 treatment through exosome-like nanoparticle prevented LPS-induced septic shock (Kim et al., 2026). The ginsenosides block glycosylation of TLR that triggered by Gram-negative bacterial LPS, which in turn downregulates cytokine storm and ROS stress in host cells (Kim et al., 2026). Furthermore, KRG is able to inhibit PCD by pyroptosis (Kim et al., 2013; Cho et al., 2023). Specifically, Ginsenosides of KRG attenuated Gram-negative bacterial LPS-mediated Caspase-11 non-canonical inflammasome and KRG extracts prevents NLRP3 inflammasome through IL-1β secretion (Kim et al., 2013; Cho et al., 2023).

Ocotillo type ginsenosides are rare ginsenosides found in natural ginsengs. Their semi-synthesis methods have continued to be developed (Zhou et al., 2013; Bi et al., 2015; Zhang et al., 2020). Chemical modifications of C3 and C12 of the furan ring are relevant for antibacterial effects of ocotillo type ginsenosides (Cao et al., 2020). Various ocotillo type ginsenoside derivatives, such as amino groups, ketones, nitric oxide (NO), and lactones, have been synthesized. Ketone substitution enhanced the antibacterial effect and NO substitution exhibits antibacterial effects through release of NO (Zhou et al., 2013; Bi et al., 2015). In addition, NO substituted derivatives are notably more effective against Gram-positive bacteria than against Gram-negative bacteria. They enhanced antibacterial effects of kanamycin and chloramphenicol against *Bacillus subtilis* and MRSA (Bi et al., 2015). Lactone-substituted derivatives disrupted bacterial cell membrane and interfere with bacterial DNA functions by inhibiting DNA polymerase and DNA topoisomerase activities (Table 2) (Zhang et al., 2020).

### 3.3 Polysaccharides and oil

Ginsan, an acidic polysaccharide found in ginseng, enhanced the phagocytosis of macrophages and decreased acute-inflammation-related cytokines and TLRs of macrophages, thereby preventing acute inflammation caused by *S. aureus* infection (Ahn et al., 2006). In addition, polysaccharides from ginseng inhibited adhesion and agglutination functions of *Porphyromonas gingivalis* to host cells (Lee et al., 2004; Lee et al., 2009). Acidic polysaccharides PG-F2 and PG-HMW from ginseng reduced adhesion activities of *Helicobacter pylori, P. gingivalis, Actinobacillus actinomycetemcomitans, Propionibacterium acnes,* and *S. aureus* (Lee et al., 2004; Lee et al., 2009). Furthermore, acidic polysaccharides from ginseng inhibited hemagglutination of erythrocytes by *P. gingivalis* (Lee et al., 2004). However, acidic polysaccharides from another medical hub, *Artermisia capillaris*, could not reduce the hemagglutination effect of *P. gingivalis* (Lee et al., 2004). The difference between these two acidic polysaccharides is the content of glucuronic acid and galacturonic acid. The content of glucuronic acid in acidic polysaccharides from ginseng was higher than that in acidic polysaccharides from *A. capillaris*, whereas the content of galacturonic acid in acidic polysaccharides from ginseng was lower. In addition, heparin (∼3,000 Da) and sucrose octasulfate sodium form (1,159 Da) showed high anti-adhesion activity to *P. gingivalis* (Lee et al., 2004). Specific negatively charged groups of the above polysaccharides are responsible for their anti-adhesion activity (Lee et al., 2004). Essential oil from *P. ginseng* leaves is composed of rich aliphatic or terpenoid compounds and a small quantity of aromatic compounds. It inhibited the growth of *S. aureus* with an inhibition zone up to 13.7 ± 0.6 mm (tetracycline control: 22.0 ± 1.5 mm) (Table 4) (Na et al., 2018).

**TABLE 4:** Reported antibacterial effects of fatty acid and polysaccharide of ginseng.

Type	Derivatives	Target bacterial species	Ref.
Fatty acid	Panaxytriol	*H. pyroli*	(Bae et al., 2001)
Polysaccharide	PG-F2	*H. pyroli, A. actinomycetemcomitans, P. acnes, P. gingivalis, P. acnes, S. aureus*	(Fan et al., 2019; Pan et al., 2023)
	PG-HMW	*P. gingivalis, A. actinomycetemcomitans, P. acnes, S. aureus*	(Pan et al., 2023)
	Ginsan	*S. aureus*	(Wajima et al., 2019)
	Acidic polysaccharide from *P. ginseng*	*P. gingivalis*	(Fan et al., 2019)

While the studies summarized above highlight that ginseng extracts and their major constituents can directly inhibit pathogenic bacteria and attenuate infection-relevant phenotypes (e.g., biofilm formation, quorum sensing, adhesion, and membrane integrity) and can also modulate host immune responses, these outcomes likely reflect only part of ginseng’s mode of action. Because many ginseng-derived compounds are biotransformed by the gut microbiota and can, in turn, reshape microbial community structure and metabolite output (e.g., bile acids and SCFAs), ginseng may influence host physiology through microbiota-dependent pathways that extend beyond the gut. Such microbiota-mediated effects are particularly relevant given the central role of gut microbial metabolites in colonization resistance, systemic immune regulation, and inter-organ communication (e.g., the gut–lung, gut–liver, and gut–brain axes), which have been implicated in diverse microbiota-associated disorders. Therefore, the next section focuses on the bidirectional interactions between ginseng and the microbiota, including microbial biotransformation of ginsenosides, prebiotic and metabolite-mediated effects, and the broader implications for diseases linked to dysbiosis.

## 4 Interactions between ginseng and microbiota

### 4.1 Gut microbiota and pre-biotic effects of ginseng on gut microbiota

Myriad bacteria constitute biological barriers in the gut microenvironment. Gut microbiota is considered a vital controlling part of the body after the brain due to their manifold involvement in physiological activities (Singh et al., 2023). The microbiota can modify or synthesize various metabolites by utilizing substances from the host. This process supplies various nutrients to the host. It also regulates physiological responses in the host. The gut microbiota can produce vital metabolites such as vitamins, bile acids, SCFAs, and essential amino acids (Sidhu and van der Poorten, 2017). Secondary bile acids transformed by gut microbiota are related to glucose tolerance and insulin resistance (Gao et al., 2022). Moreover, the gut microbiota can protect the gut from pathogen infections by inhibiting colonization of pathogenic bacteria. Indiscreet use of antibiotics can disrupt the balance of gut microbiota and allow pathogenic bacteria to colonize. *C. difficile*, a representative antibiotic-associated gut dysbiosis pathogenic bacterium, colonizes in the gut microenvironment, it can produce spores and trigger recurrent *C. difficile* infections whenever gut dysbiosis occurs (Sidhu and van der Poorten, 2017).

**FIGURE 2:**
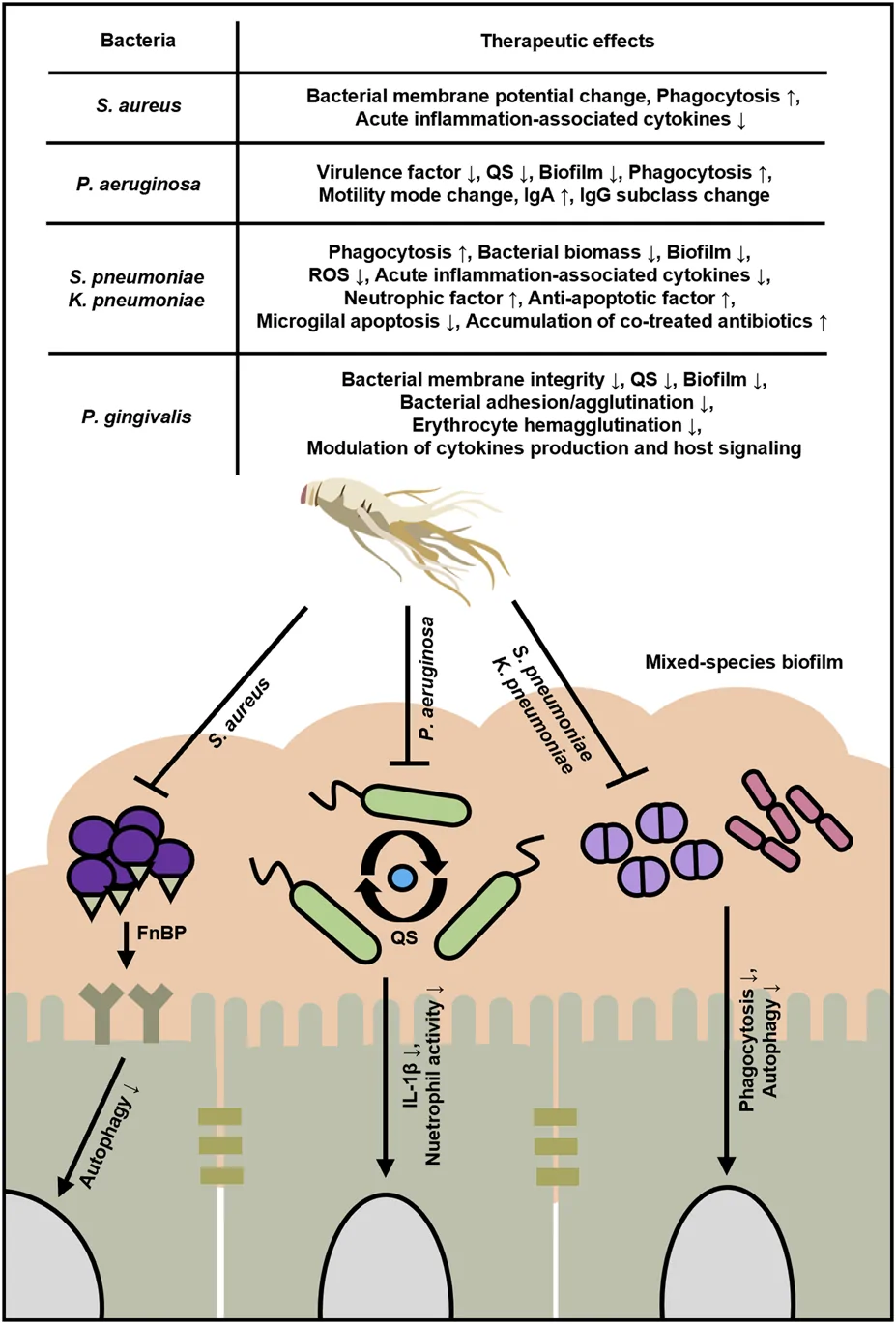
Therapeutic effects of ginseng on *P. gingivalis, P. aeruginosa, S. aureus, S. pneumoniae*, and *K. pneumoniae*. QS signal can be downregulated by ginseng treatments, leading to reduced mixed-species biofilm formation. In reduced biofilm, bacterial cells show membrane damage caused by pharmacological compounds of ginseng and become vulnerable to antibiotics. Furthermore, ginseng treatments can induce accumulation of antibiotics in bacterial cells. Notably, ginseng treatments can enhance host immune responses against pathogenic bacteria, such as upregulation of phagocytosis activity, immunoglobulin production or subclass change, and decrease of acute inflammation-associated cytokines production. FnBP: Fibronectin-binding protein, QS: Quorum sensing; Ig: Immunoglobulin; ROS: Reactive oxygen species. Figure 2 was designed with Magnific (https://www.magnific.com/) images.

What causes ginseng’s prebiotics effects for selectively inhibition effect on pathogens growth and offer of excellent environment for normal microbiota rebuilding? (Figure 2). First, indirect antibacterial effects of ginseng on pathogens triggers normal microbiota restoration. Ginseng does not kill the pathogens directly like antibiotics, but it modulates the pathogen growth through inhibition of QS or virulence factor production and enforcement of host immune system. For instance, urease production, which is essential for virulence of *H. pylori* in gastric environment, is decreased by fermented ginseng extracts (Yang et al., 2012). Inflammation and virulence of *H. pylori* decrease through host Nrf2 pathway activation by KRG extracts (Kim et al., 2022). Moreover, ginseng contributes to rebuilding normal microbiota directly.

Second, ginseng-derived compounds are good nutrients for gut health beneficial bacterial species. Ginseng is mainly metabolized by beneficial bacteria such as *Lactobacillus, Bifidobacterium*, *Eubacterium, Fusobacterium,* and *Bacteroides* (Bae et al., 2000; Bae et al., 2002a; Bae et al., 2002b; Bae et al., 2005; Quan et al., 2013; Park et al., 2001; Kim et al., 2020b; Zhang et al., 2021b). Thus, ginseng can act as a nutrient source for beneficial bacteria and increase their abundance in the gut environment. After metabolization by beneficial bacteria, biotransformed ginsenosides are involved in gut microbiota restoration. PPT type ginsenoside Rg1 can rescue disordered gut microbiota composition in dextran sulfate sodium-induced ulcerative colitis mice (Cheng et al., 2022). In addition, PPT ginsenoside Rg2 can promote the growth of *Parabacteroides distasonis*, which can decrease Th17/Treg-related inflammation in mouse gut, resulting in reduction of rheumatoid arthritis severity (Sun et al., 2023). PPD type ginsenoside Rb1 can restore gut microbiota composition by enriching *Parasutterella* spp. And reducing *Alistipes, Odoribacter, Anaeroplasma*, and *Prevotellaceae*-unclassified spp. In mice with type 2 diabetes (Zhou et al., 2023). Therefore, ginseng offers ideal environment in host for beneficial bacterial growth and pathogens elimination.

Third, ginseng treatments can change metabolite patterns of existing gut-microbiota bacterial species. Among the metabolites, SCFAs, once transported across the intestine, can enter the bloodstream and directly influence metabolism or the function of various organs (Ratajczak et al., 2019; van der Hee and Wells, 2021). Respiratory pathogens are significantly affected by the primary SCFAs. Butyrate effectively increases the survival rate of mice infected with *K. pneumoniae* (Chakraborty et al., 2017). Acetate is known to regulate *K. pneumoniae* infection by activating the free fatty acid receptor 2 (Galvão et al., 2018). Additionally, acetate has been shown to be effective against secondary lung infections. When mice infected with Influenza A virus were given a secondary pulmonary infection with *Streptococcus pneumoniae*, supplementation with acetate improved the bacterial killing activity of alveolar macrophages and increased the survival rate of the infected mice (Sencio et al., 2020). These results suggest that metabolites like SCFAs produced by gut bacteria can have a significant impact on respiratory infections. Given that ginseng is well-documented to regulate the gut microbiome by increasing the proportion of SCFA-producing bacteria, such as *Akkermansia*, further study is needed to explore the effect of ginseng on respiratory infections through the modulation of the gut microbiota (Figure 2).

### 4.2 Biotransformation of ginsenosides mediated by gut microbiota

**FIGURE 3:**
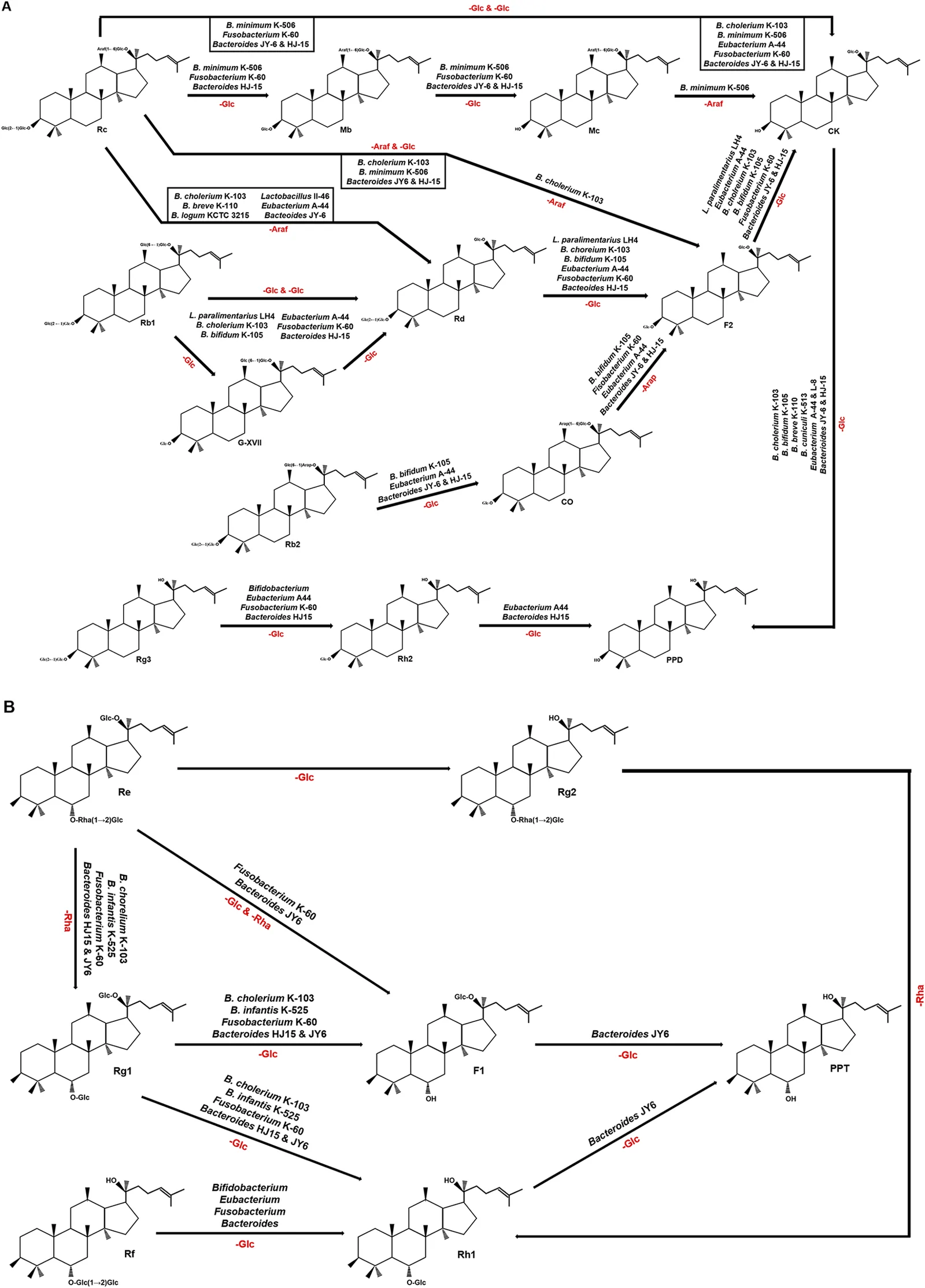
Biotransformation pathways of administrated ginsenosides involving gut microbiota. **(A)** Biotransformation pathways of PPD type ginsenosides involving gut microbiota. **(B)** Biotransformation pathways of PPT type ginsenosides involving gut microbiota. *Fusobacterium*, *Bifidobacterium*, *Lactobacillus*, *Eubacterium*, *Bacteroides* are vital for biotransformation and absorption of administrated ginsenosides. PPD: Protopanaxadiol; PPT: Protopanaxatriol.

Diverse forms of pharmaceutical ginsenosides are known to be biotransformed by the gut microbiota (Figure 3). Several enzymes involved in ginsenoside conversion have been successfully expressed and purified from bacteria, such as ginsenoside-hydrolyzing β-glucosidase from *Paecilomyces Bainer* sp. 229, *Sulfolobus solfataricus,* and *Thermotoga neapolitana* (Choi et al., 2018; Bi et al., 2019; Park et al., 2001). The gut microbiota can convert primary ginsenosides through deglycosylation, dearabinosylation, dexylosylation, and derhamonsylation at C3, C6, and C20 positions (Bae et al., 2000; Bae et al., 2002a; Bae et al., 2002b; Bae et al., 2005; Quan et al., 2013; Zhang et al., 2021a; Park et al., 2001; Kim et al., 2020a; Zhang et al., 2021b; Shao et al., 2023). *Lactobacillus, Bifidobacterium, Eubacterium, Fusobacterium*, and *Bacteroides* are core microbiota for biotransformation of ginsenosides (Bae et al., 2000; Bae et al., 2002a; Bae et al., 2002b; Bae et al., 2005; Quan et al., 2013; Park et al., 2001; Kim et al., 2020b; Zhang et al., 2020). Ginsenoside Rb1 and Rb2 can be converted to Rd and compound O (CO) through deglycosylation at the C20 position, respectively (Bae et al., 2000; Quan et al., 2013; Park et al., 2001). Ginsenoside Rc can be converted to Rd, Rg3, and Mb through dearabinofusylation, deglycosylation/dearabinofusylation at the C20 position and deglycosylation at the C3 position, respectively (Bae et al., 2002a; Bae et al., 2002b). Ginsenoside Rd, CO, and Mb can be transformed into F2 and Mc, which are key intermediates for interchange to a high pharmaceutical compound ginsenoside CK through additional deglycosylation and dearabinofusylation at C3 and C20 positions, respectively (Kim et al., 2020b; Zhang et al., 2020; Shao et al., 2023). In a healthy human gut, *Alistipes finegoldii* and *Parabacteroides merdae* are mainly involved in the conversion of ginsenoside CK to PPD (Huang et al., 2023). PPT-type ginsenoside Re can be transformed to Rg1, Rg2, and F1 through deglycosylation and derhamnosylation at C6 and C20 positions (Bae et al., 2005). Another PPT-type ginsenoside Rf can be transformed to Rh1 through deglycosylation at the C6 position (Bi et al., 2019). Furthermore, ginsenoside Rg1 and Rg2 can be altered to Rh1 through deglycosylation at the C20 position and derhamnosylation at the C6 position (Bae et al., 2005).

### 4.3 Effects of ginseng on various diseases associated with gut microbiota

**FIGURE 4:**
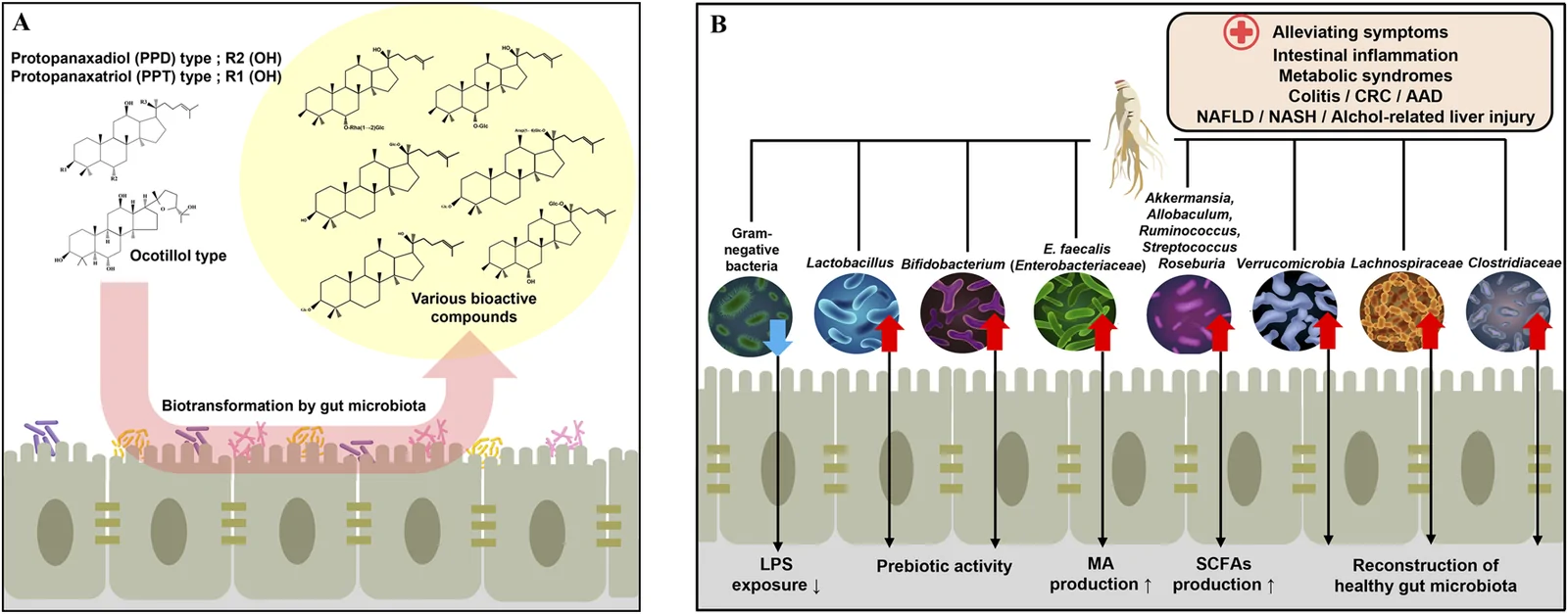
Ginsenoside biotransformation by gut microbiota and restoration of collapsed gut microbiota by ginseng treatments and beneficial effects in gut-other organs axes. **(A)** Scheme of ginsenoside biotransformation by gut microbiota. **(B)** Ginseng treatment can restore collapsed gut microbiota compositions and alleviate gut microbiota dysbiosis-associated diseases (red cross symbol). Ginseng treatment is able to decrease the abundance of Gram-negative bacteria, which produce a pathogenic factor LPS, in the gut microbiota. In contrast, prebiotic bacteria, secondary metabolites producing bacteria, and strict anaerobic bacteria are increased by ginseng treatment. In conclusion, these gut microbiota restorations can improve symptoms of various abnormal gut microbiota-associated diseases. CRC: colitis-associated colorectal cancer; AAD: Antibiotics-associated diarrhea**;** NAFLD: Nonalcoholic fatty liver disease; NASH: Non-alcoholic steatohepatitis; LPS: Lipopolysaccharide; MA: Myristoleic acid; SCFA: Short-chain fatty acid. Figure 4 was designed with Magnific (https://www.magnific.com/) images.

Various studies highlighted that microbiota dysbiosis is closely related with disease development. Notably, numerous phyla localized at gut environments and exhibited lots of advantageous effects in healthy microbiota state (Aggarwal et al., 2023). Also, gut microbiota interacts with gut localized host cells including immune cells through microbial messengers (Fan and Pedersen, 2021). However, gut microbiota dysbiosis due to erroneous diet habits or antibiotic abuse triggers abnormal signaling pathways (Lu et al., 2024). Even pathogenic bacterial species can take up the empty gut microenvironments and produce lots of virulence factors (Lu et al., 2024). Representatively well-studied gut-microbiota dysbiosis-associated diseases are intestinal inflammation, obesity, antibiotic-associated diarrhea (AAD), colitis/colitis-associated colorectal cancer (CRC), and nonalcoholic fatty liver disease (NAFLD) (Lu et al., 2024). Development of these diseases is correlated with several taxa alteration in the gut microenvironments. Thus, restoration of gut-microbiota to healthy state is vital for prevention or alleviation of symptoms. To date, many trials for gut microbiota restoration, such as fecal microbiota transplantation, phage therapy, pre-/pro-/synbiotics, and microbiota mimetics, are studied and developed (Gulliver et al., 2022). Nevertheless, these methods have several limitations for widespread application because of difficulty of modulation complex gut-microbiota, temporary effects, and high cost (Gulliver et al., 2022). Ginseng, a natural herb adjuvant, can be a safe and inexpensive substitute for restoration of gut-microbiota or prevention of gut microbiota dysbiosis (Table 5). How ginseng is different from other therapeutic compounds is that ginseng harbors diverse therapeutic compounds including ginsenosides, polysaccharides, and oil. These compounds are modified (biotransformed) to various therapeutic derivatives by gut microbiota, which in turn they work in combination on maintaining/restoration of healthy gut microbiota and interactions of gut-other organs axes (Figure 4A).

**TABLE 5:** Reported modulatory effects of ginseng on various gut-microbiota dysbiosis-associated diseases.

Related disease	Treated ginseng compounds	Microbiota modulation	Ref.
Intestinal inflammation	Polysaccharides	Gram-negative bacteria ↓	(Wang et al., 2021a)
	Polysaccharides/Ginsenosides mixture	Shiga-toxin producing bacteria ↓/*Lactobacillus* ↑/*Bifidobacterium* ↑	(Zhou et al., 2021)
	Ginsenoside Rh4	*Firmicutes* : *Bacteroidetes* ↓	(Bai et al., 2021)
Obesity	Whole ginseng extract	*E. faecalis* ↑/MA-producing bacteria ↑	(Quan et al., 2020)
	Ginsenoside R1, Rb1, rd, re, rf, and Rg1	*A. muciniphila* ↑/*P. distasonis* ↑	(Zhou et al., 2021)
	Ginseng extracts of KRG	*Verrucomicrobia* ↑/*Proteobacteria* ↑/*Deferribacteres* ↑/*Bacteroidetes* ↓	(Lee et al., 2021)
	Ginseng extracts of KRG without ginsenosides	*Verrucomicrobia* ↑/*Proteobacteria* ↑/*Bacteroidetes* ↓	(Lee et al., 2021)
Metabolic syndrome	Ginseng extracts of KRG	No significant change	(Seong et al., 2021)
AAD	Fermented ginseng	*E. faecium* ↑/*L, murinus* ↑/*B. infantis* ↑/*Enterobacteriaceae* ↑	(Qu et al., 2021)
	Polysaccharides	*Lactobacillus* ↑/*Bacteroides* ↓	(Li et al., 2019)
Colitis/CRC	Red ginseng extracts	*Lactobacillus* : *E. coli* ↑	(Guo et al., 2015)
	American ginseng extracts	*Verrucomicrobia* ↓/*Firmicutes* ↑	(Wang et al., 2016; Wang et al., 2018)
		*Porphyromonadaceae* ↓	(Wang et al., 2018; Natividad et al., 2015; Baxter et al., 2016)
	Polysaccharides	Shannon index ↑/Gram-negative bacteria ↓	(Wang et al., 2023)
IBD	Ginsenoside	*Akkermansia* ↑/*Peptostreptococcaceae* ↑/*Jeotagalicoccus* ↑	(Wang et al., 2021a)
NAFLD	KRG	*Akkermansia* ↑	(Hong et al., 2021)
Alcohol-induced liver injury	Fermented ginseng extracts	*Akkermansia* ↑/*Allobaculum* ↑/*Ruminococcus* ↑/*Lactobacillus* ↑/*Streptococcus* ↑/*Roseburia* ↑	(Fan et al., 2019)

KRG: korean red ginseng; AAD: Antibiotics-associated diarrhea; CRC: Colitis-associated colorectal cancer; IBD: inflammatory bowel disease; NAFLD: Non-alcoholic fatty liver disease.

#### 4.3.1 Intestinal inflammation

Intestinal inflammation is one of the microbiota-associated diseases. It occurs due to over-activation of the intestinal immune system through exposure to multifarious bacterial virulence factors such as lipopolysaccharide (LPS) and toxins (Wang et al., 2021a). Treatment with polysaccharides from *P. ginseng* can influence the restoration of microbiota after exposure to antibiotics. For instance, a mixture of polysaccharides derived from American ginseng and ginsenosides Rh4 has demonstrated significant effectiveness in restoring gut microbiota in mice and rats with induced intestinal inflammation or immune disorders caused by cyclophosphamide, antibiotics, or dextran sulfate sodium (DSS) (Wang et al., 2021b; Zhou et al., 2021; Bai et al., 2021). Treatment with polysaccharides of *P. ginseng* reduced the abundance of Gram-negative bacteria, thereby down-regulating abnormal LPS-induced over-activation of TLR4-mediated immune responses (Wang et al., 2023). Treatment with polysaccharides/ginsenosides mixture decreased the abundance of Shiga-toxin producing bacteria, but increased the abundance of *Lactobacillus* and *Bifidobacterium*, resulting in promotion of mucosal immunity (Zhou et al., 2021). Ginsenoside Rh4 reduced the ratio of *Firmicutes* to *Bacteroidetes*, which leads to the production of SCFAs for maintaining the integrity of the intestinal barrier (Figure 4B) (Bai et al., 2021).

#### 4.3.2 Obesity and metabolic syndrome

Obesity is a salient disease associated with gut microbiota dysbiosis. Ginseng treatments enable the host to counteract the progression of obesity. Treatment with whole ginseng extracts increased the abundance of *Enterococcus faecalis*, a myristoleic acid (MA)-producing bacterium, in obese mice (Quan et al., 2020). Elevation of MA can inhibit adiposity through activation of brown adipose tissue and high accumulation of beige fat (Quan et al., 2020). Notably, treatment with ginsenosides can alter gut microbiota compositions in obese mice. Treatment with ginsenosides (R1, Rb1, Rd, Re, Rf, and Rg1) from *P. notoginseng* increased the abundance of *Akkermansia muciniphila* and *P. distasonis* (Xu et al., 2020). The above bacteria increased in the gut commensal microbiota can lessen host adiposity (Xu et al., 2020). Indeed, treatment with ginsenosides-containing extracts of KRG (whole extracts) increased *Verrucomicrobia* and *Proteobacteria* as shown in healthy mice (Figure 4B). In contrast, treatment with extracts without ginsenosides results in a different alteration pattern of gut microbiota (*Deferribacteres*) in high-fat diet-induced obese mice (Lee et al., 2021). Thus, ginsenosides are salient pharmacological compounds for restoring obesity-induced gut microbiota dysbiosis. Treatment with KRG extracts to metabolic syndrome mice did not show a significant alteration of a gut microbiota pattern (Seong et al., 2021). However, KRG treated group exhibited lower homeostatic model assessment insulin resistance (HOMA-IR) value and total cholesterol and low-density lipoprotein levels in the serum were significantly elevated compared with those in the placebo group (Seong et al., 2021).

#### 4.3.3 Antibiotics-associated diarrhea (AAD)

Main cons of antibiotics are alteration of gut microbiota composition and over-growth of pathogenic bacteria in an empty gut. Although diarrhea can induce the clearance of pathogens from the intestine, it also disrupts gut microbiota (Young and Schmidt, 2004). Therefore, reconstruction of gut microbiota after diarrhea is vital for maintaining a healthy gut environment. Administration of fermented ginseng at a concentration of 0.5 g/kg/day to AAD rats restored total bacterial contents to levels in non-AAD rats. Four standard bacterial species, *Enterococcus faecium, Lactobacillus murinus, Bifidobacterium infantis,* and *Enterobacteriaceae*, were recovered from collapsed gut microbiota, except for *Bacteroides* (Qu et al., 2021). In particular, treatment with fermented ginseng (0.5 and 1.0 g/kg/day) increased the abundance of *Bifidobacterium* more in AAD rats than in non-AAD rats (Qu et al., 2021). Treatment of AAD mice with ginseng polysaccharides increased *Lactobacillus*, but significantly decreased *Bacteroides* compared to ginseng polysaccharides non-treated AAD mouse (Li et al., 2019). The above two studies revealed that ginseng treatment could trigger rapid restoration of normal gut microbiota after AAD (Figure 4B) (Li et al., 2019).

#### 4.3.4 Colitis and colitis-associated colorectal cancer (CRC)

Colitis is a disease caused by inflammation in the colon. Colitis is able to be developed to CRC for 10–20 years and the risk of CRC progression is 2 to 3-fold higher in ulcerative colitis (UC) patients (Wang et al., 2021a; Li et al., 2022). Several bacteria species are linked with CRC such as *Bacteroides fragilis*, *Escherichia coli*, *E. faecalis*, *F. nucleatum, Porphyromonas* spp., *Prevotella* spp., and *Streptococcus gallolyticus* (Rebersek, 2021). Therefore, regulation of dysbiosis gut-microbiota is crucial for CRC inhibition and ginseng is able to be beneficial by its gut-microbiota restoration effect. Administration of red ginseng extracts to ulcerative colitis rats could increase the abundance of *Lactobacillus* and inhibit the growth of *E. coli* (Guo et al., 2015). Red ginseng also exhibited an anti-inflammatory effect in colon tissues as confirmed by microscopic observation of reduced malondialdehyde content and myeloperoxidase activity. Thus, red ginseng could rescue colon tissues from inflammation through its anti-inflammatory activity. It also aids in the reconstruction of collapsed *Lactobacillus* to *E. coli* ratio (Guo et al., 2015). Feeding American ginseng extracts to azoxymethane (AOM)/DSS-induced colitis or CRC mice can restore the microbiota pattern similar to that of non-colitis/CRC mice (Wang et al., 2016). In AOM/DSS-induced colitis/CRC groups, the ratio of *Verrucomicrobia* to *Firmicutes* was increased, rapidly (Wang et al., 2016; Wang et al., 2018). In contrast, the amount of increased ratio was significantly reduced in the group fed American ginseng (Wang et al., 2016; Wang et al., 2018). In CRC patients, the number of gut bacteria is generally lower than that of the healthy control group, with notably reduced levels of *Clostridiaceae* and *Lachnospiraceae* (Wang et al., 2018). In addition, anti-inflammatory bacteria such as *Firmicutes* were upregulated in the group fed with American ginseng extracts (Wang et al., 2018). Furthermore, CRC-inducing bacteria *Porphyromonadaceae* were downregulated in the same manner as in the non-CRC group after feeding with American ginseng extracts (Figure 4B) (Wang et al., 2018; Natividad et al., 2015; Baxter et al., 2016). Not only whole extracts of ginseng, but polysaccharides or ginsenosides of ginseng can affect positively to abnormal microbiota which is involved in colitis (Zhou et al., 2021; Wang et al., 2023). Polysaccharides of *P. ginseng* to DSS-induced colitis rats exhibit higher Shannon index (degree of microbial community diversity) in polysaccharides treated group than model group (Wang et al., 2023). Polysaccharides of *P. ginseng* decreased the abundance of Gram-negative bacteria which produce virulence factor LPS (Wang et al., 2023). Treatment of ginsenoside fraction to inflammatory bowel disease (IBD) model rats increased abundances anaerobes such as *Akkermansia,* *Peptostreptococcaceae* *,* and *Jeotgalicoccus*. These alterations of microbiota composition can modulate metabolite patterns such as SCFAs, glutathione, and amino acids (tryptophan, histidine, and alanine) (Wang et al., 2021b).

#### 4.3.5 Liver-related diseases

Microbiota compositions can affect liver-related diseases such as NAFLD and alcohol-related liver injury. Values of alanine aminotransferase (ALT) and aspartate aminotransferase (AST) activity as indicators of NAFLD were ameliorated in KRG-treated patients with NAFLD (Hong et al., 2021). The abundance of *Lactobacillus* and intestine integrity-promoting bacteria *Akkermansia* were increased in KRG-treated patients with NAFLD compared to the placebo group (Hong et al., 2021). In general, the genera number of altered gut microbiota is significantly correlated with AST or ALT activity (Hong et al., 2021). The overall number of ALT-correlated genera was higher in the placebo group than in the KRG-treated group. However, a higher number of genera negatively correlated with ALT activity was observed in the KRG-treated group (Hong et al., 2021). Furthermore, treatment of NAFLD mice with ginsenosides altered the gut microbiota pattern to relieve NAFLD indicator values (Hong et al., 2021). Eighteen operational taxonomic units (OTUs) of the altered gut microbiota due to ginsenoside treatment were found to exhibit positive or negative correlations with NAFLD indicator values (Liang et al., 2021). Microbiota reconstruction effects of ginseng treatment in mice with alcohol-induced liver injury have been reported (Fan et al., 2019). Treatment with ginseng extracts fermented by *Lactobacillus fermentum* KP3, which contain increased levels of rare ginsenosides, increased the abundance of SCFAs-producing bacteria such as *Akkermansia, Allobaculum, Ruminococcus, Lactobacillus, Streptococcus,* and *Roseburia* in mice with alcohol-induced liver injury (Fan et al., 2019). SCFAs provide nutrients to intestinal epithelial cells and exhibit anti-inflammatory and anti-tumor effects (Fan et al., 2019). Notably, *Akkermansia* can reduce intestinal permeability and lead to prevention of bacterial LPS penetration into the bloodstream and inhibition of excessive immune responses (Figure 4B) (Fan et al., 2019). Recently, inhibition mechanism of lipid peroxidation and ferropotosis pathway in accord with modulation of gut microbiota by ginsenoside Rd was elucidated (Liu et al., 2025). Ginsenoside Rd regulated the gut microbiota composition to enhance lipid metabolism, which in turn inhibited ferroptosis in liver cells and consequently reduced the induction of metabolism-associated fatty liver disease (Liu et al., 2025).

## 5 Synergic effects of ginseng with other therapeutic agents and novel therapies

Plenty of the above studies have revealed that crude ginseng extracts, purified ginsenosides, and acidic polysaccharides from ginseng can be utilized as therapeutic adjuvants. Their various immune modulation and antibacterial effects can be enhanced by co-treatment with other therapeutic agents or vaccines. Ginsenoside 20(S)-Rh2 increased the accumulation rate of ciprofloxacin (CIP) in *S. aureus* cell bodies, resulting in highly concentrated CIP that can easily kill bacterial cells in mice (Zhang et al., 2014). In addition, an improvement effect of sperm motility and reduced germ cell apoptosis have been observed in male rats with bacterial-induced epididymo-orchitis after co-administration of KRG extracts with ciprofloxacin (CIP) (Eskandari et al., 2016).

Ginseng is an attractive synergistic adjuvant with various therapeutic agents. Ginseng polysaccharides and ginsenosides from *P. quinquefolius* showed synergistic effects in restoring gut structure and microbiota composition (Zhou et al., 2021). Under cyclophosphamide-induced immunosuppression, normalization of T cell and B cell proliferation, an increase of NO production in peritoneal macrophages, and elevation of serum IL-12/IF-γ levels were observed after pidotimod treatment with acidic polysaccharide of *P. ginseng* co-administration (Du et al., 2008). Rhubarb and carbenoxolone treatment with ginseng improved neurological deficiency score, cerebral infarction, and regulation of Cx43/AQP4 expression in ischemia/reperfusion-injured rats (Yang et al., 2019). Co-treatment of hydroxyzine, primrose oil, and cyclosporine with KRG extracts significantly decreased the severity, dermal inflammation, and IL-17 levels in atopic dermatitis mice (Woo et al., 2021).

Moreover, ginseng exhibits synergistic effects when co-treated with other plant extracts. Various plant extracts combined with KRG extracts improved amnestic effect in scopolamine-induced memory impairment mice (Wee et al., 2005). Co-treatment with anionic molecules from *Codium fragile* and aged garlic extracts with red/black ginseng extracts increased immune responses in immune-suppressed mice and decrease lipid biomarkers of obesity in high-fat diet-induced obese mice (Kim et al., 2020a; Jiang et al., 2021). Piperine co-administration increased the permeability of ginsenoside Rh2 and decreased the metabolism of Rh2 so that serum IL-2 level was increased and maintained for an extended period (Zhao-Hui et al., 2018). Based on these synergistic effects of ginseng, several studies have investigated herb-drug interactions using ginseng extracts and ginsenosides. Red ginseng extracts and ginsenoside Rc did not affect to organic anion transporters (OATs) activity. Thus, pharmacokinetics of valsartan, a high blood pressure and heart failure drug, is not changed significantly (Jeon et al., 2020). However, KRG extracts increased expression of cytochrome P450 enzymes, multiple drug resistance transporters, and OATs involved in drug absorption, transport, metabolism, and excretion (Kim et al., 2018a). The metabolism pattern of hypnotic-sedative drug midazolam was not significantly changed by KRG extract co-treatment (Kim et al., 2018b). However, the exposure ratio of antihistamine drug fexofenadine to the entire body was decreased by KRG extracts (Kim and Yang, 2018). Thus, when using ginseng extracts or ginseng compounds in conjugation with other therapeutic agents, it is essential to meticulously control their concentration.

Despite ginseng, which has above various beneficial effects, is treated as Generally Recognized as Safe (GRAS) by The Food and Drug Administration (FDA), several limitations and adverse effects are still remained. Current evidences regarding the antibacterial properties and gut microbiota modulation of ginseng remains largely restricted to *in vitro* and preclinical animal models. Moreover, existing human clinical investigations referenced herein fall short of the robustness provided by large-scale and multi-center trials. Consequently, there is a compelling need for subsequent studies to undertake comprehensive clinical evaluations to bridge this gap in the current understanding of ginseng’s therapeutic potential. Representative adverse effect is a sensation of heat*,* represented by ginseng-abused syndrome (GAS) which occurred by alteration of Na^+^-K^+^-ATPase index (Ran et al., 2022). Furthermore, ginsenosides, which are major active compounds of ginseng, decrease blood thinner agent, Wafarin (Dong et al., 2017). In addition to the aforementioned adverse effects, ginseng exhibits application limitations, including interindividual variability in efficacy influenced by host health status and gut microbiota composition. Nevertheless, current research remains insufficient to comprehensively address these adverse effects and limitations. Accordingly, further quantitative and mechanistic studies are required to support the safe and effective clinical application of ginseng.

## 6 Conclusion

Pathogenic bacterial co- or secondary infections remain a significant health concern, while antibiotic-based treatments are often limited by toxicity, disruption of beneficial microbiota, and the emergence of multi-drug resistance. Therefore, alternative therapeutic strategies that combine antimicrobial and immune-regulating functions are needed. Ginseng, which contains diverse bioactive compounds such as ginsenosides and acidic polysaccharides, exhibits antibacterial, immunomodulatory, and microbiota-restoring effects. Because it acts as a multi-component therapeutic rather than a single-molecule drug, ginseng can provide synergistic benefits, including suppression of pathogenic bacteria and restoration of dysbiotic gut microbiota. Thus, ginseng represents a promising non-antibiotic approach for preventing or managing infections associated with microbiota imbalance.
